# Tuberculosis Detection in Chest Radiographs Using Spotted Hyena Algorithm Optimized Deep and Handcrafted Features

**DOI:** 10.1155/2022/9263379

**Published:** 2022-10-06

**Authors:** Seifedine Kadry, Gautam Srivastava, Venkatesan Rajinikanth, Seungmin Rho, Yongsung Kim

**Affiliations:** ^1^Department of Applied Data Science, Noroff University College, Kristinasand 4612, Norway; ^2^Department of Electrical and Computer Engineering, Lebanese American University, Byblos, Lebanon; ^3^Artificial Intelligence Research Center (AIRC), College of Engineering and Information Technology, Ajman University, Ajman, UAE; ^4^Department of Mathematics and Computer Science, Brandon University, Brandon, R7A 6A9, Canada; ^5^Research Center for Interneural Computing, China Medical University, Taichung 40402, Taiwan; ^6^Department of Computer Science and Engineering, Saveetha School of Engineering, Saveetha Institute of Medical and Technical Sciences, Chennai 602105, Tamil Nadu, India; ^7^Department of Industrial Security, Chung-Ang University, Seoul, Republic of Korea; ^8^Department of Technology Education, Chungnam National University, Daejeon, Republic of Korea

## Abstract

Lung abnormality in humans is steadily increasing due to various causes, and early recognition and treatment are extensively suggested. Tuberculosis (TB) is one of the lung diseases, and due to its occurrence rate and harshness, the World Health Organization (WHO) lists TB among the top ten diseases which lead to death. The clinical level detection of TB is usually performed using bio-medical imaging methods, and a chest X-ray is a commonly adopted imaging modality. This work aims to develop an automated procedure to detect TB from X-ray images using VGG-UNet-supported joint segmentation and classification. The various phases of the proposed scheme involved; (i) image collection and resizing, (ii) deep-features mining, (iii) segmentation of lung section, (iv) local-binary-pattern (LBP) generation and feature extraction, (v) optimal feature selection using spotted hyena algorithm (SHA), (vi) serial feature concatenation, and (vii) classification and validation. This research considered 3000 test images (1500 healthy and 1500 TB class) for the assessment, and the proposed experiment is implemented using Matlab®. This work implements the pretrained models to detect TB in X-rays with improved accuracy, and this research helped achieve a classification accuracy of >99% with a fine-tree classifier.

## 1. Introduction

In the healthcare industry, there is a heavy diagnostic burden because of the steady increase in disease incidence in humans due to various reasons. The burden of disease detection can be reduced in hospitals by developing and implementing automated disease detection systems using artificial intelligence (AI) [1–5].

The lungs are one of the vital internal organs, and an infection in the lungs can cause severe illness, including death [6–8]. Tuberculosis (TB) is one of the severe lung diseases caused by Mycobacterium tuberculosis (*M. tuberculosis*), and it can cause severe breathing problems in human patients. Therefore, it is imperative to detect and treat *tuberculosis* in a timely manner. It is also a communicable illness that will affect a human quickly and easily if one has a weak immune system.

A recent report by World Health Organization (WHO) lists TB as one of the top 10 causes of death globally and the foremost reason for death from a solitary infectious agent [9]. This report also confirms that, in 2019, TB caused 1.4 million deaths worldwide, and this report estimated that ten million people would be diagnosed with TB. Most people infected with TB (>*t* 90%) are adults, and the infection rate in males is higher than in women. Increased TB rate in a country is due to poverty, which causes financial distress, susceptibility, marginalization, and bias in TB-infected people. Furthermore, this report also verifies that about a quarter of the world's population is infected with TB. Usually, TB is curable and preventable when diagnosed in its early phase, and >85% who develop TB can be completely recovered with a 6-month drug regimen [10, 11].

The clinical level diagnosis of TB is usually performed with various clinical tests, including the bio-images. The infected lung section is typically recorded using computed tomography (CT) and radiographs (X-ray). The recorded image is then examined using a computer algorithm or by an experienced doctor to identify the harshness of TB. The former research on TB detection confirms that early diagnosis is essential to reduce the disease burden; hence, the researchers suggest several automated diagnostic procedures [12, 13]. In literature, the detection of TB with chest X-ray is widely discussed due to its clinical significance. Several machine learning (ML) and deep learning (DL) procedures are developed and employed to assess chest X-ray pictures. The DL-supported scheme helps to achieve a better detection accuracy compared to the ML, and hence, the DL-supported TB detection is considered in this research. The proposed research proposes a TB detection framework using the pretrained DL scheme, which implements combined segmentation and classification to achieve better detection, as discussed in [14]. The earlier work by Rahman et al. [14] implemented UNet for the segmentation and pretrained DL schemes for the classification. In the earlier work, the performance of VGG16 is not discussed, and hence, this research attempted the detection of TB using the VGG-UNet-based technique. The different stages of this framework consist of (i) image collection and resizing, (ii) implementation of pretrained VGG-UNet to segment the lung section from X-ray, (iii) collection of deep features (DF), (iv) local-binary-pattern (LBP) generation using different weights and LBP feature extraction, (v) spotted hyena algorithm (SHA) based DF and HF reduction, (vi) generating a new feature section with the serial concatenation of features, and (vii) binary classification and validation.

In this work, 3000 test images (1500 healthy and 1500 TB) are collected from the dataset provided by Rahman et al. [14, 15]. Initially, every test image is resized to 224 × 224 × 3 pixels, and the converted images are then evaluated using the pretrained VGG-UNet. UNet is a well-known convolutional neural network (CNN)-based encoder-decoder assembly, and the enhancement of this scheme is already reported in the literature. The enhancement methods, such as VGG-UNet [16] and ResNet [17], are already employed in which the encoder section is modified using the DL scheme. In the considered VGG-UNet, the well-known VGG16 architecture is considered to implement the encoder-decoder assembly, and the earlier work on this scheme can be accessed from [4]. In this work, the encoder section provides the necessary DF, and the decoder section supplies the segmented lung section, which is then considered to extract HF. The optimal value of DF and HF is then identified using SHA, and then, a serial concatenation is considered to combine these optimal features (DF + HF). This feature vector is then considered to validate the performance of the binary classifier with a 5-fold cross-validation, and the employed scheme helped to achieve a classification accuracy of 98.73% with the fine-tree classifier.

The main contribution of this research includes the following:

- Execution of CNN-based joint segmentation and classification is implemented using VGG16
- LBP pattern generation with various weights is presented
- SHA-based feature selection and serial feature concatenation is discussed

Other sections are arranged as follows: Section 2 shows earlier related work, Section 3 demonstrates methodology, and Sections 4 and 5 present the experimental outcome and conclusion of this research.

## 2. Related Research

Automated disease detection schemes are developed to reduce the diagnostic burden in hospitals, and most of these schemes also support the decision-making and treatment planning processes. In the literature, several ML and DL schemes are discussed to identify the TB from chest X-rays with the help of benchmark and clinically collected images. Every procedure aims to get better detection accuracy. This section summarizes chosen procedures employed to examine the X-ray, and the necessary information is presented in Table 1.

The research by Rahman et al. [14] employed a combined segmentation and thresholding concept to improve disease detection performance. This work employed the proposed technique on 7000 images (3500 healthy and 3500 TB class) and presented a detailed examination using various pretrained CNN methods in the literature. With an experimental investigation, the proposed work confirmed that joint segmentation and classification help to get a better disease diagnosis. With this motivation, the proposed work of this research also adopted the joint segmentation and classification concept to examine the TB from the database provided by Rahman et al. [15]. In the earlier work, the VGG16 was not employed for the segmentation and classification task. Hence, the proposed research work adopted the VGG-UNet scheme for the investigation, in which the VGG16 acts as the encoder unit. The experimental outcome of this study confirms that the proposed scheme worked well on the chest X-ray database and helped to achieve a classification accuracy of >99% with the fine-tree classifier.

## 3. Methodology

This research division shows the scheme developed to identify the TB by joint segmentation and classification task. First, the necessary test pictures are collected from a benchmark image database represented by Rahman et al. [15], and after the collection, every image is resized to a dimension of 224 × 224 × 3 pixels. After the resizing task, every picture is evaluated by the VGG-UNet. Then, the encoder section presents the necessary DF, and the final layer (SoftMax) of the decoder section provides the binary form of the segmented lung section. The outcome of the encoder unit provides a DF of value, which is then reduced by 1 × 1 × 1024 using a chosen dropout rate (2 dropout layers with 50% dropout value to reduce 1 × 1 × 4096 to 1 × 1 × 1024), and these features are further reduced using the SHA to get the DF of a chosen dimension. The binary image obtained at the decoder section is then combined with its original test image to extract the lung section. The necessary LBP features are extracted from the extracted lung section, and these features are further reduced with SHA. Finally, a serial concatenation is then implemented to get DF + HF, and these features are then chosen to test and validate the performance of the developed system on the considered database.

**Figure 1:**
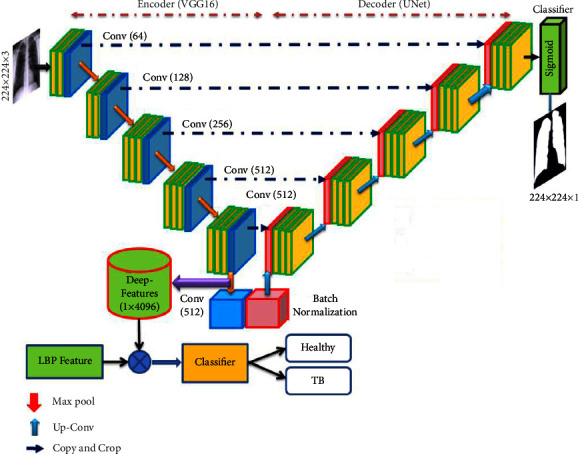
Joint segmentation and classification implemented for TB detection using X-ray.

The performance of the proposed scheme is tested using (i) DF alone and (ii) SHA-optimized DF + HF. During this assessment, the SoftMax-based binary classification is employed, and later, other binary classifiers existing in the literature are considered for testing the performance of the proposed scheme. The various stages presented in this scheme are shown in Figure 1. The concatenated feature is employed in this work to classify the X-ray images into healthy/TB classes.

### 3.1. Image Dataset

**Figure 2:**
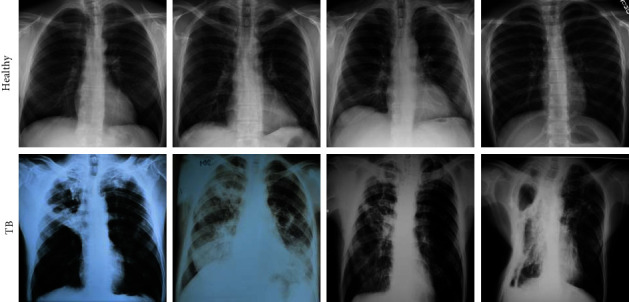
Sample X-ray images of healthy/TB class.

The merit of the automated disease diagnosis is then tested and verified using the clinically grade or benchmark medical data. In this research, the chest X-ray images considered by Rahman et al. [15] are adopted. From this dataset, 3000 images are collected to assess which 1500 images belong to the healthy group and the remaining 1500 with TB traces. Every collected image is resized to 224 × 224 × 3 pixels (approved size for VGG16). Of the total images, 70% (1050 images) are considered to train the developed scheme, and the remaining 30% (450 images) are considered for validation. The information about the test images is shown in Table 2, and the sample test images for the healthy/TB class are presented in Figure 2.

### 3.2. Pretrained VGG-UNet

Deep-learning-supported medical data assessment is a commonly employed technique, and most of these approaches are adopted to implement automatic segmentation and classification operations [26–28]. The CNN-based segmentation using the traditional UNet [29] and SegNet [30] is employed in the literature to extract and evaluate the disease-infected section from various modality medical images. The limitation of traditional CNN segmentation schemes is rectified by enhancing its performance using the pretrained DL schemes. The DL schemes are considered to form the encoder and decoder section, which supports the feature extraction and segmentation for medical images of a chosen dimension. In this work, the pretrained VGG16 is then considered to implement the VGG-UNet scheme, and the necessary information about this architecture can be found in [4, 31].

Initially, the considered VGG-UNet is trained using X-ray images with the following tasks.

- Predictable augmentation (rotation and zoom) to increase the number of training images
- Assignment of learning rate as 1*e*-4 for better accuracy
- Training with linear dropout rate (LDR) and Adam optimization

During this task, other vital parameters are assigned as follows: total iteration = 2000, total epochs = 50, dropout rates in the fully connected layer = 50%, and the final layer is the SoftMax unit with 5-fold cross-validation.

### 3.3. Feature Extraction

This section presents the outline of the DF and HF extraction procedure.

#### 3.3.1. Deep Feature

The necessary deep features from the proposed scheme are extracted from the encoder section (VGG16) of the VGG-UNet. This section offered a feature vector of dimension 1 × 1 × 4096, and it is then passed through three fully connected (FC) layers with a dropout rate of 50% to get a reduced feature vector of dimension 1 × 1 × 1024. This feature is the DF, which is then considered to classify the X-ray images using a chosen binary classifiers. In this work, the classification task is executed using the conventional DF and the DF optimized using the SHA. The experimental outcome of this study confirms that the proposed work helped to get better classification accuracy with optimized DF compared to the conventional DF.

#### 3.3.2. Handcrafted Feature

The HF is considered in ML-based automatic disease detection systems, and in this work, the HF is obtained using LBP of various weights as discussed in [32]. The various procedures to extract the HF from the chosen X-ray are as follows: the implemented VGG-UNet helps to extract the lung section in binary form. This binary image is then combined with its original test image to get the necessary lung section without the artifacts. After getting the lung image, the necessary LBP pattern is generated by assigning its weights as *W*=1,2,3 an d 4, and from these images, the necessary LBP features with dimension 1 × 1 × 59 are extracted, and the extracted features are then optimized using the SHA.

**Figure 3:**
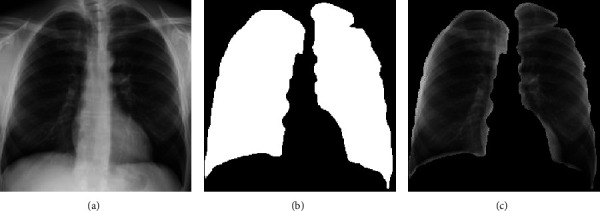
The result achieved with VGG-Unet and the extracted lung section. (a) Test image, (b) binary image, (c) section to be examined.

**Figure 4:**
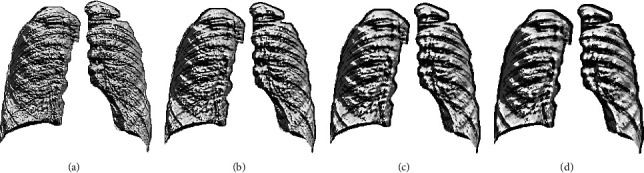
Generated LBP pattern of X-ray for various weights. (a)*W* = 1, (b)*W* = 2, (c)*W* = 3, (d)*W* = 4.

Figure 3 depicts the procedure to remove the artifact. Figures 3(a) and 3(b) show the sample test image and the extracted binary region by the decoder section of VGG-UNet. When Figures 3(a) and 3(b) are combined (pixel-wise multiplication), then we get Figure 3(c), which is the lung section without the artifact. This section is then considered to get the LBP pattern with various weights. The generated LBP pattern of a sample image is presented in Figure 4, in which Figures 4(a)–4(d) depict the outcome for the chosen weights. Every image provides 59 number of one-dimensional (1D) features, and the total features obtained with LBP are 236 features (59 × 4) which are then reduced using the SHA to avoid the overfitting difficulty in X-ray classification. Other information related to this task can be found in the earlier research works [4, 16].

### 3.4. Feature Reduction with Spotted Hyena Algorithm

Metaheuristic algorithms (MA) are adopted in the literature to find the finest solution for various real-world problems. The earlier works related to medical image assessment confirm that the MA is widely adopted in various image examination works, such as thresholding, segmentation, and feature selection [33, 34]. The MA-based feature selection procedure is already discussed in various ML and DL techniques, and this procedure helps to get the finest feature vector, which avoided the overfitting problem during the automated classification. The MA-based feature selection can be used as an alternative technique for the traditional feature reduction procedures discussed in [35].

In this work, the feature reduction task is implemented for both the DF and HF using the SHA. It is a nature-motivated procedure invented in 2017 by mimicking the hunting events found in spotted hyena (SH) packs. The SH are the skillful animal that hunts as a pack, and this operation consists of the following stages: (i) choice making and following the prey, (ii) chasing the prey, (iii) surrounding the prey, and (iv) killing. The arithmetical replica developed by Dhiman and Kumar [36, 37] considered all constraints to improve the converge capability of the SHA. A similar kind of algorithm, known as the Dingo optimizer, is also developed and implemented by Bairwa et al. [38].

**Figure 5:**
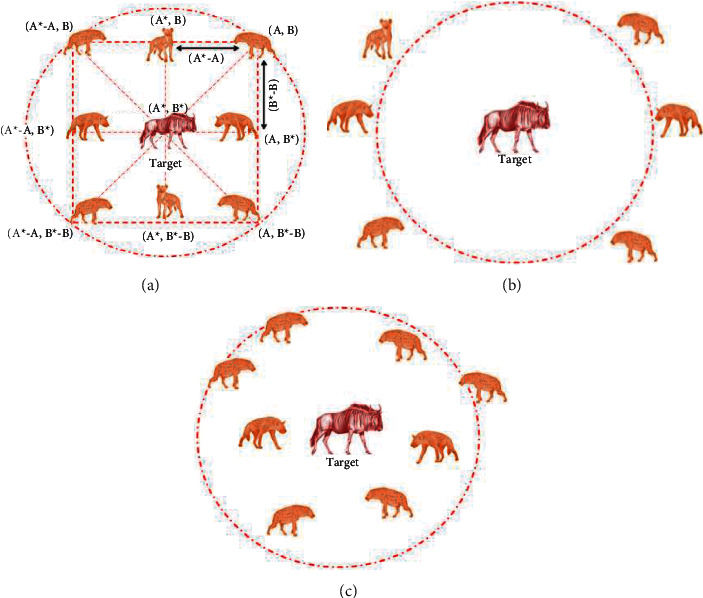
Various stages of SHA. (a) Encircling, (b) hunting, (c) attacking.

The various stages of the SHA are depicted in Figure 5, in which Figures 5(a)–5(c) present the operations, such as identifying and tracking the prey as in Figure 5(a), tracking and encircling the prey depicted in Figure 5(b), and hunting as presented in Figure 5(c). This operation is as follows: the leader in pack identifies the prey, and the leader and its pack will chase it till it is tired. When the prey is tired, the leader and its group will encircle the prey as depicted in Figure 5. In this context, every group member will adjust their location concerning the prey. This process is depicted in the figure using notation A and B. This adjustment is carried out in the algorithm using mathematical operations such as multiplication and subtraction.

The encircling process is mathematically represented as follows:

(1) $$\begin{array}{c} {\overset{⟶}{D}}_{h}=\left|\overset{⟶}{B}.{\overset{⟶}{P}}_{p}\left(x\right)-\overset{⟶}{P}\left(x\right)\right|\text{,} \end{array}$$

(2) $$\begin{array}{c} \overset{⟶}{P}\left(x+1\right)={\overset{⟶}{P}}_{p}\left(x\right)-\overset{⟶}{E}.{\overset{⟶}{D}}_{h}\text{,} \end{array}$$

where ${\overset{⟶}{D}}_{h}$ = distance among the hyena and prey, *x* = current iteration, ${\overset{⟶}{P}}_{p}$ = position vector of prey, and $\overset{⟶}{P}$ = position vector of hyena.

The coefficient vectors $\overset{⟶}{B}$ and $\overset{⟶}{E}$ are computed as follows:

(3) $$\begin{array}{c} \overset{⟶}{B}=2.R\overset{⟶}{d_{1}}\text{,} \end{array}$$

(4) $$\begin{array}{c} \overset{⟶}{E}=2\overset{⟶}{h}.R\overset{⟶}{d_{2}}-\overset{⟶}{h}\text{,} \end{array}$$

(5) $$\begin{array}{c} \overset{⟶}{h}=5-\left(\text{Iteration}*\left(\frac{5}{Iter_{\max}}\right)\right)\text{,} \end{array}$$

where Iter_max_ = maximum iterations assigned, $\overset{⟶}{h}$ = *a* linearly decreasing value from 5 to 0 insteps of 0.1, $ℜ\overset{⟶}{d_{1}}$ and $ℜ\overset{⟶}{d_{2}}$ = random number [0,1] number

In this figure, (*A*, *B*) are the hyena, and it will adjust its location towards the prey (*A∗*, *B∗*) based on the values of Eqns. (3) to (5).

In the hunting stage, the hyena pack will move close to the prey and proceed for the attack. This phase is represented as follows:

(6) $$\begin{array}{c} {\overset{⟶}{D}}_{h}=\left|\overset{⟶}{B}.{\overset{⟶}{P}}_{h}-\overset{⟶}{P_{k}}\right|\text{,} \end{array}$$

(7) $$\begin{array}{c} {\overset{⟶}{P}}_{k}={\overset{⟶}{P}}_{h}-\overset{⟶}{E}.{\overset{⟶}{D}}_{h}\text{,} \end{array}$$

(8) $$\begin{array}{c} {\overset{⟶}{C}}_{h}=\overset{⟶}{P_{k}}+\overset{⟶}{P_{k+1}}+\cdots +\overset{⟶}{P_{k+N}}\text{,} \end{array}$$

where ${\overset{⟶}{P}}_{h}$ = leader which moves closer to prey and $\overset{⟶}{P_{k}}$ = positions of other hyenas in the pack, and *N* = total hyenas in the pack.

In the attacking phase, the hyena moves and attacks the prey, other hyenas in the group also follow the same technique, and the group attach will kill the prey. When the prey is dead, every hyena in the pack is on or nearer to the prey. This process is the convergence of the chosen agents towards the optimal location as in (7).

(9) $$\begin{array}{c} \overset{⟶}{P}\left(x+1\right)=\frac{\overset{⟶}{C_{h}}}{N}\text{,} \end{array}$$

where $\overset{⟶}{P}\left(x+1\right)$ is the best position, in which every hyena of the pack converges. In this work, the SHA is initiated with the following parameters: number of hyena (agent) = *N* = 20, search dimension (D) = 2, Iter_max_ = 2000 and stopping criteria = maximization of Cartesian distance (CD) between features or Iter_max_.

**Figure 6:**
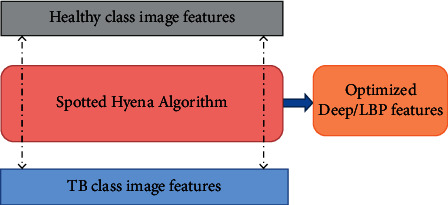
The SHA-based feature selection process.

The feature reduction with SHA is graphically depicted in Figure 6, and from this procedure, it is clear that this task selects the image features of the healthy/TB class based on the maximal CD, and this procedure is already discussed in earlier works [5, 34, 39]. As depicted in Figure 6, the SHA-based feature selection is separately employed to identify the optimal features, and after getting these features, it is then serially concatenated, and the concatenated features are then considered for classifier training and validation.

The number of DF and HF available for the optimization is depicted in the following equations:

(10) $$\begin{array}{c} DF_{V\text{GG}\left(1\times 1\times 1024\right)}=VGG_{\left(1,1\right)},VGG_{\left(1,2\right)},\cdots ,VGG_{\left(1,1024\right)}\text{,} \end{array}$$

(11) $$\begin{array}{c} LBP1_{w1\left(1\times 1\times 59\right)}=W1_{\left(1,1\right)},W1_{\left(1,2\right)},\cdots ,W1_{\left(1,59\right)}\text{.} \end{array}$$

(12) $$\begin{array}{c} LBP2_{w2\left(1\times 1\times 59\right)}=W2_{\left(1,1\right)},W2_{\left(1,2\right)},\cdots ,W2_{\left(1,59\right),} \end{array}$$

(13) $$\begin{array}{c} LBP3_{w3\left(1\times 1\times 59\right)}=W3_{\left(1,1\right)},W3_{\left(1,2\right)},\cdots ,W3_{\left(1,59\right)}\text{,} \end{array}$$

(14) $$\begin{array}{c} LBP4_{w4\left(1\times 1\times 59\right)}=W4_{\left(1,1\right)},W4_{\left(1,2\right)},\cdots ,W4_{\left(1,59\right)}\text{,} \end{array}$$

(15) $$\begin{array}{c} HF_{\left(1\times 1\times 236\right)}=LBP1+LBP2+LBP3+LBP4\text{.} \end{array}$$

During the feature reduction process, the DF in Eqn. (10) and HF in (8) are examined by the SHA, and the reduced features are then serially concatenated to get a hybrid feature vector (DF + HF). In this work, the SHA helps to reduce the DF to 1 × 1 × 427and HF to 1 × 1 × 166, and these features are then combined as in Eqn. (16) to get the new feature vector.

(16) $$\begin{array}{c} DF+HF_{\left(1\times 1\times 593\right)}=DF_{\left(1\times 1\times 427\right)}+HF_{\left(1\times 1\times 166\right)}\text{.} \end{array}$$

The feature presented in (9) is then considered to train and test the classifiers considered in this study. The various binary classifiers considered in this research include SoftMax, Naïve-Bayes (NB), random forest (RF), decision tree (DT) variants, K-nearest neighbors (KNN) variants, and SVM with linear kernel [40–43].

### 3.5. Performance Validation

The merit of an automated disease detection system is to be verified by computing the necessary performance values. In this work, the measures obtained from the confusion matrix are considered to confirm the eminence of the proposed scheme. These measures include true positive (TP), false negative (FN), true negative (TN), false positive (FP), accuracy (ACC), precision (PRE), sensitivity (SEN), specificity (SPE), and negative predictive value (NPV). The mathematical expressions of these values are presented in the following equations [42–46]:

(17) $$\begin{array}{c} ACC=\frac{TP+TN}{TP+TN+FP+FN,}\text{,} \end{array}$$

(18) $$\begin{array}{c} PRE=\frac{TP}{TP+FP}\text{,} \end{array}$$

(19) $$\begin{array}{c} SEN=\frac{TP}{TP+FN}\text{,} \end{array}$$

(20) $$\begin{array}{c} SPE=\frac{TN}{TN+FP}\text{,} \end{array}$$

(21) $$\begin{array}{c} NPV=\frac{TN}{TN+FN}\text{.} \end{array}$$

## 4. Results and Discussion

This part of the research presents the present research's investigational outcome. This work is executed using a workstation; Intel i7 2.9 GHz processor with 20 GB RAMS and 4 GB VRAM equipped with Matlab®.

Initially, the pretrained VGG-UNet scheme is trained using resized chest X-ray images till it extracts the lung section with better accuracy. After the training, its segmentation performance is tested using test images, and the outcome is recorded. Then, the extracted section is combined with the original image to extract the lung section without the artifact, and the necessary LBP is generated when the HF is extracted. Similarly, the necessary DF is extracted from the encoder section (VGG16), and these features are then passed through the fully connected layers to get a feature vector of size 1 × 1 × 1024. This procedure is similar to employing a traditional VGG16 scheme, and this feature is initially considered to classify the images with a SoftMax classifier, and the necessary performance is then recorded.

**Figure 7:**
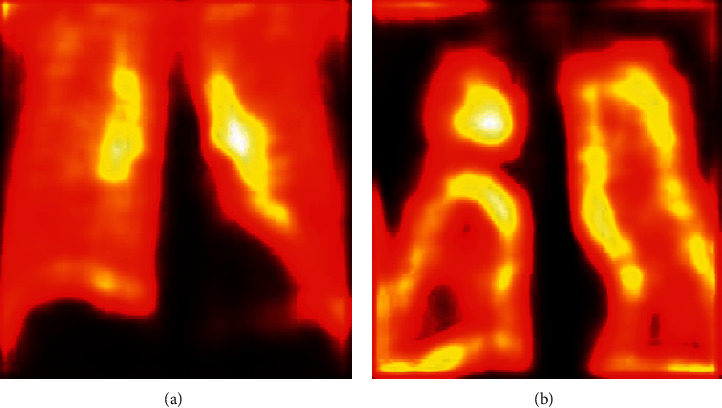
Sample test images obtained the convolution operation. (a) Normal, (b) TB.

**Figure 8:**
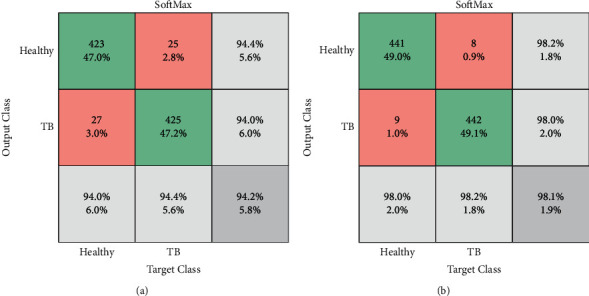
Confusion matrix attained with traditional and optimized features. (a) DF with SoftMax, (b) DF + HF with SoftMax.

During the convolutional operation, the layers of the VGG-UNet help to recognize the necessary image features to support the necessary feature extraction and segmentation. The sample test image textures identified during a convolutional operation are presented in Figure 7. Figures 7(a) and 7(b) depict the hot color map image obtained for healthy and TB class sample images, respectively. After extracting the necessary deep features from the test images (with a VGG16-like scheme), the necessary classification task is implemented using the SoftMax classifier with a 5-fold cross-validation. The achieved results are presented in Table 3. This table confirms that when the DF vector of dimension 1 × 1 × 1024 is considered, SoftMax provided a classification accuracy of 94.22%. This procedure is then repeated using the SHA-selected DF + HF presented in (9), and the achieved confusion matrix (CM) is presented in Figure 8.  Figure 8(a) shows the CM of the DF case, and Figure 8(b) shows the CM of optimized DF + HF, and this confirms that the accuracy achieved with the proposed method is superior to the traditional technique. Hence, the performance of the proposed scheme is then confirmed with various binary classifiers using the DF + HF.

**Figure 9:**
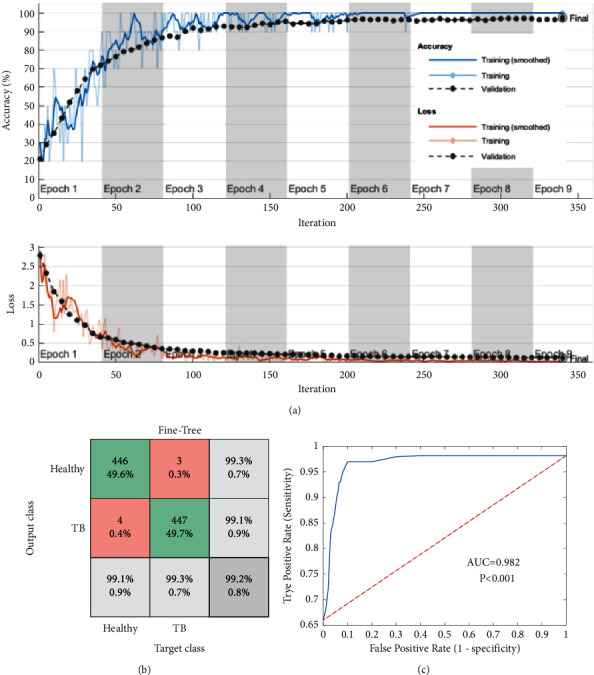
Investigational outcome of the fine-tree classifier with DF + HF. (a) Convergence, (b) confusion matrix, (c) ROC.

Figure 9 presents the experimental outcome achieved with the RF variant and the fine-tree classifier.  Figure 9(a) depicts the convergence of the search, and Figures 9(b) and 9(c) show the CM and the receiver operating characteristic (ROC) curve, respectively. The results achieved with other chosen classifies are presented in Table 4. This confirms that the classification accuracy of the fine tree is >99%, which confirms its merit over other techniques. In order to verify the performance of the proposed scheme, its best result is compared with the results of Rahman et al. [14] and confirmed that the proposed joint segmentation and classification scheme with SHA-selected DF + HF help to achieve a better outcome compared to the earlier works.

**Figure 10:**
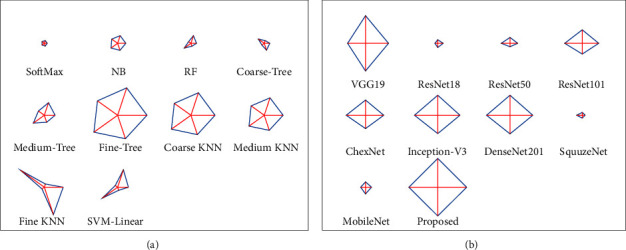
Overall performance of TB detection system demonstrated with glyph plot. (a) Results with DF + HF, (b) comparison with earlier work.

Table 4 confirms that the result of the fine tree is better than other binary classifiers, and the coarse KNN also helped to achieve a classification accuracy of 90% compared to other techniques. This confirmed that the optimized DF + HF supported classification helps to get a better overall result, as presented in Figure 10.  Figure 10(a) shows the glyph plot for the overall performance of binary classifiers, and the pattern covering a maximum area is considered superior. The comparison of the fine-tree classifier with earlier works is presented in Figure 10(b), which confirms its superiority over other classifiers. Figure 10(b) compares the ACC. PRE, SEN, and SPE of the earlier research by Rahman et al. [14] with the fine-tree result, and this comparison confirms that the proposed system's outcome is better.

This research implemented a joint segmentation and classification scheme to detect TB from chest X-rays with better accuracy. The main limitation of the proposed scheme is that it considered the artifact-removed image for getting the necessary HF from the LBP images. In the future, the LBP can be combined with other HF existing in the literature. Furthermore, the performance of the proposed scheme can be tested and verified with other benchmark chest X-ray images with various lung abnormalities.

## 5. Conclusion

In humans, TB is a severe disease that widely affects the lungs, and early diagnosis and treatment will help to reduce the severity. Furthermore, the timely detection and recommended medication will help to cure the TB completely. Due to its significance, a considerable number of research works are performed by researchers to support the automated diagnosis. This research aims to develop and implement a joint segmentation and classification scheme with the help of a pretrained VGG-UNet scheme. The VGG-UNet system consists of an encoder-decoder assembly, in which the encoder helps to get the necessary DL features as in the traditional VGG16 system, and the decoder associated with the SoftMax classifier helps to extract the binary form of the lung image. This work considered the LBP pattern of the lung image to extract the necessary HF. This work considered the LBP with varied weights and helped to get a1D feature vector of size 236. The extracted DF and the HF are then optimized using the SHA, and these features are then serially united to get the concatenated features vector (DF + HF). This feature vector is then considered for testing and validating the performance of the binary classifiers using 5-fold cross-validation. The experimental outcome of this study confirmed that the binary classification with the fine-tree classifier helped to achieve an accuracy of >99% for the considered chest X-ray images. This result is then compared and validated with the result of other DL methods available in the literature. This research confirmed the merit of the proposed DF + HF-based TB detection from the chest X-ray images. In the future, this scheme can be enhanced with other HF available in the literature. Furthermore, the performance of the proposed scheme can be tested and validated with other chest X-ray image datasets available in the literature.

## Figures and Tables

**Table 1:** Summary of automated TB detection schemes employed to examine X-ray images.

Reference	Developed procedure
Rajaraman and Antani [18]	A customized DL system is proposed to examine the Shenzhen CXR pictures, and the proposed system provided an accuracy of 83.7%. However, this work confirms that implementing a customized DL approach is complex and time-consuming

Hwa et al. [19]	Examination of TB from X-ray using ensemble DL system and canny-edge detection is implemented and achieved better values of accuracy (89.77%), sensitivity (90.91%), and specificity (88.64%). However, the implementation of canny-edge detection along with the ensemble DL scheme needs a larger image preprocessing task, and it will increase the detection time

Wong et al. [20]	The development of a customized DL technique called TB-Net is proposed, and this work helped to achieve better performance measures, such as accuracy (99.86%), sensitivity (100%), and specificity (99.71%). This research also proposes a customary model, which is relatively more complex than the pretrained models

Hooda et al. [21]	Seven convolutional layers and three fully connected layer-based customized DL method are proposed for TB detection and achieved a classification accuracy of 94.73%

Rohilla et al. [22]	This work employed the conventional AlexNet and VGG16 methods to examine the X-ray images and attained an accuracy of >81%

Nguyen et al. [23]	X-ray diagnosis performance of pretrained DL schemes is presented, and the employed technique helped to provide better TB recognition

Afzali et al. [24]	The contour-based silhouette descriptor technique is employed to detect TB, and the selected features provided an accuracy of 92.86%

Stirenko et al. [25]	The CNN-based disease diagnosis with lossless and lossy data expansion is employed, and the proposed method offers a better TB diagnosis with X-ray pictures

Rahman et al. [14]	Implementation of combined CNN segmentation and categorization is presented to identify TB from X-ray images. This work implemented the classification task with and without segmentation and achieved a TB detection accuracy of 96.47% and 98.6%, respectively. This work also presented a detailed evaluation methodology for TB detection using various pretrained DL methods

**Table 2:** Chest X-ray image dataset information.

Class	Dimension	Images		
		Total	Training	Validation
Healthy	224 × 224 × 3	1500	1050	450
TB	224 × 224 × 3	1500	1050	450

**Table 3:** Classification result achieved with DF alone for a 5-fold cross-validation.

Classifier	TP	FN	TN	FP	ACC (%)	PRE (%)	SEN (%)	SPE (%)	NPV (%)
Fold 1	418	32	414	36	92.44	92.07	92.89	92.00	92.82
Fold 2	421	29	419	31	93.33	93.14	93.56	93.11	93.53
Fold 3	420	30	424	26	93.78	94.17	93.33	94.22	93.39
Fold 4	423	27	425	25	94.22	94.42	94.00	94.44	94.03
Fold 5	421	29	423	27	93.78	93.97	93.56	94.00	93.58

**Table 4:** Sample results attained with the implemented UNet scheme.

Classifier	TP	FN	TN	FP	ACC (%)	PRE (%)	SEN (%)	SPE (%)	NPV (%)
SoftMax	441	9	442	8	98.11	98.22	98.00	98.22	98.00
NB	442	8	443	7	98.33	98.44	98.22	98.44	98.23
RF	441	9	443	7	98.22	98.44	98.00	98.44	98.01
Coarse tree	442	8	442	8	98.22	98.22	98.22	98.22	98.22
Medium tree	442	8	444	6	98.44	98.66	98.22	98.67	98.23
Fine tree	446	4	447	3	99.22	99.33	99.11	99.33	99.11
Coarse KNN	445	5	446	4	99.00	99.11	98.89	99.11	98.89
Medium KNN	444	6	445	5	98.78	98.88	98.67	98.89	98.66
Fine KNN	447	3	442	8	98.78	98.24	99.33	98.22	99.32
SVM linear	441	9	445	5	98.44	98.88	98.00	98.89	98.02

## Data Availability

The X-ray images considered in this research work can be accessed from https://ieee-dataport.org/documents/tuberculosis-tb-chest-x-ray-database.
